# Enhancing Clinical Insights: New Radiographic Grading for Lumbar Facet Joint Degeneration, A Reliability Study

**DOI:** 10.1002/jsp2.70035

**Published:** 2025-01-07

**Authors:** Xiaowen Liu, Zhenyu Zhu, Shouyu He, Mingjian Sun, Tianyi Zhao, Lei Liu, Haoyang Shi, Yang Hou, Guodong Shi

**Affiliations:** ^1^ The Department of Orthopaedic Surgery, Changzheng Hospital Second Military Medical University Shanghai China; ^2^ The Department of Spine Surgery, the First People's Hospital of Huzhou First Affiliated Hospital of Huzhou University Huzhou China

**Keywords:** degeneration, facet joint, imaging

## Abstract

**Background:**

Lumbar facet joint diseases can often lead to reduced work efficiency and increased medical costs. As a primary imaging tool in orthopedics, X‐rays offer numerous advantages. However, there is no consensus on the classification of lumbar facet joints based on X‐ray imaging.

**Methods:**

This study was conducted for 356 patients between November 2017 and September 2023. A senior radiologist and a senior orthopedic surgeon discussed and established a grading system for lumbar facet joints based on both X‐ray and MRI findings. Two double‐blind attending spinal surgeons were asked to evaluate and grade the included cases. The intra‐rater reliability and inter‐rater reliability were evaluated by assessing the weighted kappa (κw) coefficient.

**Results:**

The level of inter‐rater reliability of MRI for complete agreement was good for *Doctor A* (κw = 0.792) and *Doctor B* (κw = 0.869). The inter‐rater reliability coefficient for grading lumbar facet joint degeneration based on the radiograph was as follows: for *Doctor A* (κw = 0.702, session 1; κw = 0.847, session 2) and *Doctor B* (κw = 0.714, session 1; κw = 0.828, session 2). Moreover, the level of intra‐rater reliability based on X‐ray for complete agreement was excellent for *Doctor A* (κw = 0.843) and *Doctor B* (κw = 0.836).

**Conclusion:**

We propose a new grading system for lumbar facet joint degeneration based on X‐rays, which serves as a supplement to the Weishaupt and Pathria classifications. This grading system aims to provide clinicians with a more comprehensive understanding of lumbar facet joint degeneration, allowing for the use of a broader range of diagnostic tools to evaluate facet joint degeneration from multiple perspectives. We believe that this grading system can be valuable in assessing potential anatomical changes related to the facet joint, thereby informing modifications to surgical techniques and procedural steps.

## Graphical abstract

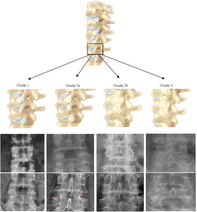

## 1 Introduction

Approximately 70% to 85% of adults will experience low back pain at some point in their lives, making it one of the most common health problems and a leading cause of disability worldwide [1]. For many individuals, low back pain lasts more than 12 weeks, progressing to chronic low back pain (CLBP) [2, 3]. The prevalence of CLBP ranges from 11% to 23% among people with low back pain [4, 5]. As a result of a prolonged loss of function, CLBP causes reduced productivity at work and higher medical expenses [6]. Although persistent low back pain can be caused by a variety of factors, including intervertebral disk, facet joint, and ligamentous dysfunction, facet joint dysfunction is considered a critical one in the development of CLBP [7]. Literature reports indicate that 15%–45% of CLBP is related to small joint dysfunction [8, 9].

Facet joints are synovial joints located between the superior and inferior articular processes of neighboring vertebrae [10, 11]. These joints feature hyaline cartilage surfaces and receive innervation from the medial branch nerves. Facet joint arthropathy refers to degenerative arthritis characterized by the breakdown of cartilage, reduction in facet joint space, and the development of osteophytes [12]. A community‐based population study discovered that 89% of adults aged 65 and older exhibited moderate to severe lumbar facet joint arthropathy [13]. Furthermore, the study revealed an age‐related increase in both the prevalence and severity of lumbar facet joint degeneration. Several risk factors for lumbar facet joint degeneration were proposed, such as higher body mass index (BMI), sagittal orientation of the facet joints, poor spinal extensors, and higher values of pelvic incidence [14].

A fundamental consensus has been established regarding the classification of degeneration of lumbar intervertebral facet joints through MRI and CT scans, particularly using the Weishaupt and Pathria classification [15]. However, both inspection equipment exhibit drawbacks such as inconvenience, high cost, and high difficulty in instrument operation, hindering their widespread adoption [16, 17, 18]. Through extensive literature review, we found that there is currently no widely accepted grading system based on X‐rays for lumbar facet joint degeneration. X‐rays, as a basic diagnostic tool for assessing the severity of lumbar spine pathology, offer advantages such as convenience, low radiation exposure, and cost‐effectiveness [19]. This led us to consider proposing a new grading system for lumbar facet joint degeneration based on X‐rays. This system would serve as a supplement to the Weishaupt and Pathria classifications, enabling clinicians to gain a more comprehensive understanding of lumbar degeneration and assess facet joint degeneration from multiple perspectives using a broader range of diagnostic tools. Therefore, building on the grading system for osteoarthritis proposed by Professors Kellgren and Lawrence [20], and taking into account the unique anatomical structure and degenerative patterns of the facet joints in the human spine, we have developed a new grading system. This system is designed to evaluate lumbar facet joint degeneration as observed in radiological examinations.

## 2 Materials and Methods

### 2.1 Sample Size Determination

In order to calculate the minimum sample size for the study, we conducted a pre‐study analysis of lumbar intervertebral joint degeneration based on radiography using PASS 15 software. This analysis demonstrates that in order to achieve a statistical difference of significance level less than 0.05, at least 355 patients need to be included in this study.

### 2.2 Inclusion and Exclusion Criteria

All patients met the following inclusion criteria: (1) Age range from 18 to 86 years old; (2) Complete preoperative X‐ray and MRI examination.

Exclusion criteria: (1) Scoliosis, Cobb angle > 20°; (2) Vertebral axial rotation: On coronal radiographs, the distance between the inner edge of the bilateral pedicle and the center point of the spinous process is inconsistent, or Nash‐Moe rotation being equal or greater than grade I on the standing anteroposterior (AP) radiograph [21, 22]; (3) Lack of preoperative X‐ray or MRI examination; (4) The interval between preoperative X‐ray and MRI examinations is more than 1 month; (5) Previous lumbar spine internal fixation surgery; (6) Lumbar spondylolisthesis > 5 mm, with severe dislocation of facet joints; (7) Intravertebral bone cement implantation; (8) Severe spinal joint disease (e.g., mandatory spondylitis); (9) spinal trauma; (10) Spinal tumors; (11) Infectious diseases of spine.

### 2.3 Study Population

**FIGURE 1:**
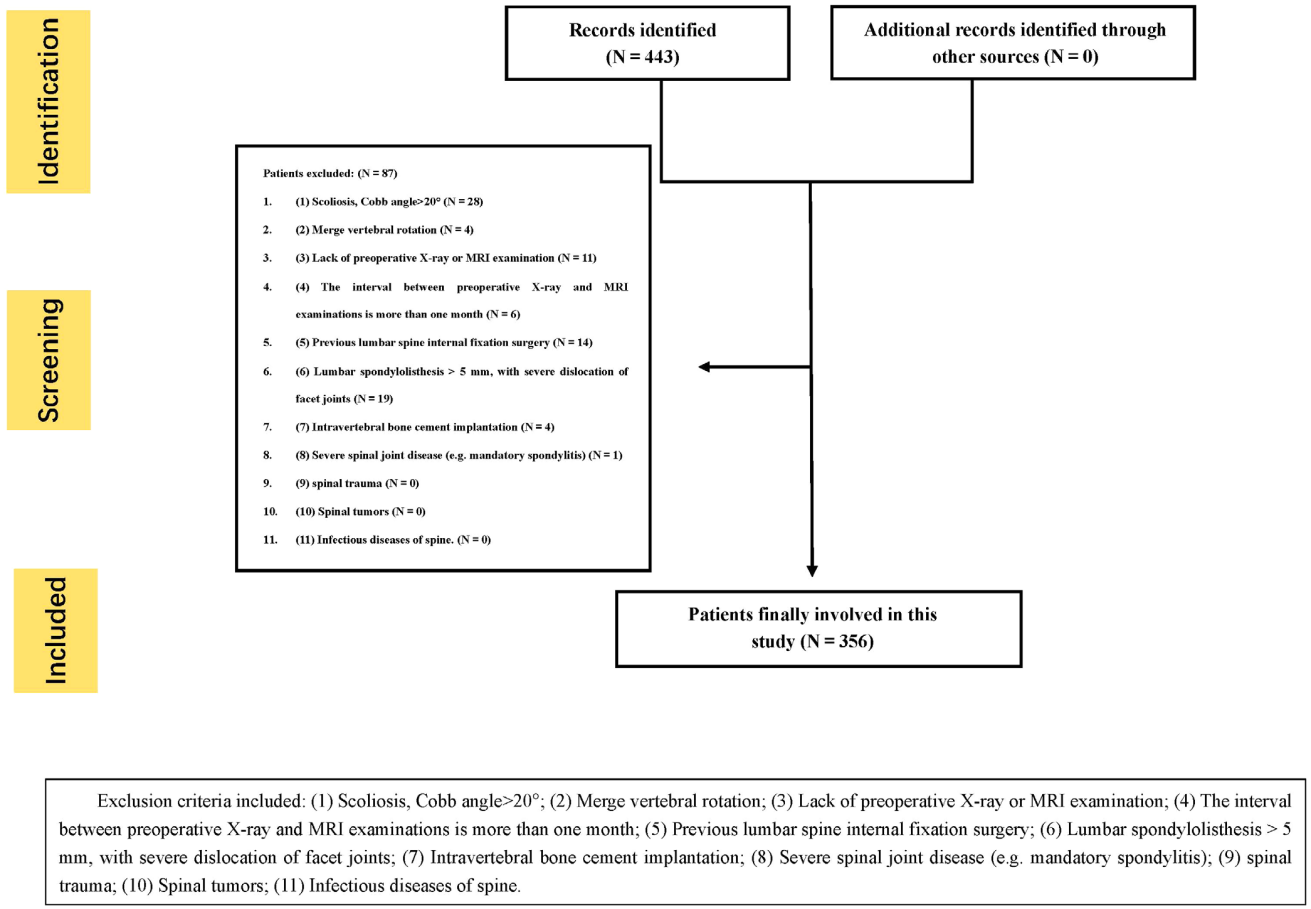
Study flow chart.

Patients aged 18 years who required imaging for the evaluation of lumbar facet joint degeneration between November 2017 and September 2023 were included. A total of 443 consecutive patients were initially enrolled. Of these, 28 patients had scoliosis, four patients had vertebral rotation, 11 patients lacked preoperative X‐ray or MRI examination, six patients had an interval of more than 1 month between preoperative X‐ray and MRI examinations, 14 patients had previously undergone lumbar spine internal fixation surgery, 19 patients had lumbar spondylolisthesis, four patients had undergone bone cement implantation surgery, and one patient with severe spinal joint disease were excluded (total *n* = 87) (Figure 1). The remaining 356 patients (mean age, 58.6 ± 12.1 years; men: women, 158:198 [mean ages, 56.8 ± 12.8 and 60.1 ± 11.4 years, respectively]) were eventually included in our analysis.

### 2.4 Imaging Equipment and Position

Each subject underwent X‐ray imaging of the lumbar spine (*L1‐S1, SIEMENS, Fluorospot Compact FD, Germany, field of view* (FOV): approximately 200 mm, *image matrix*: 2600 × 1600, *image zoom*: 1.00) in a standing position. MR imaging was performed on lumbar of patients using a clinical 1.5 T MR system (*GE MEDICAL SYSTEMS, SIGNA EXCITE, USA*). Imaging parameters were as follows: The MRI sequences included axial T1‐weighted (*Time of Echo* (TE): 9 ms, *Time of Repetition* (TR): 420 ms, *Echo train*: 3; *image zoom*: 1.00, *slice thickness*: 4 mm, *flip angle*: 90°), axial T2‐weighted (TE: 100 ms, TR: 2500 ms, *Echo train*: 26; *image zoom*: 1.00, *slice thickness*: 4 mm, *flip angle*: 90°), and transverse T2‐weighted MRI sequences (TE: 110 ms, TR: 3200 ms, *Echo train*: 24, *image matrix*: 256 × 256, *image zoom*: 1.00, *slice thickness*: 3 mm, *flip angle*: 90°).

All patients underwent lumbar spine X‐rays in the standing AP positioning. According to standard radiographic techniques, keep the patient upright facing the radiation source, and ensure that the midsagittal plane coincides with the midline of table top and the Bucky [23]. The vertical central ray is centered on the L3 midline and located at the level of the inferior costal margin. To ensure consistency in the shooting position, patients were accompanied by our research staff and guided into position with their assistance.

All imaging protocols were standardized. The radiographs were taken by the same experienced radiologist and stored using the Picture Archiving and Communication System (PACS). Subsequent measurements and evaluations of facet joints were completed on this system.

### 2.5 A New Grading System for Lumbar Facet Joints

**FIGURE 2:**
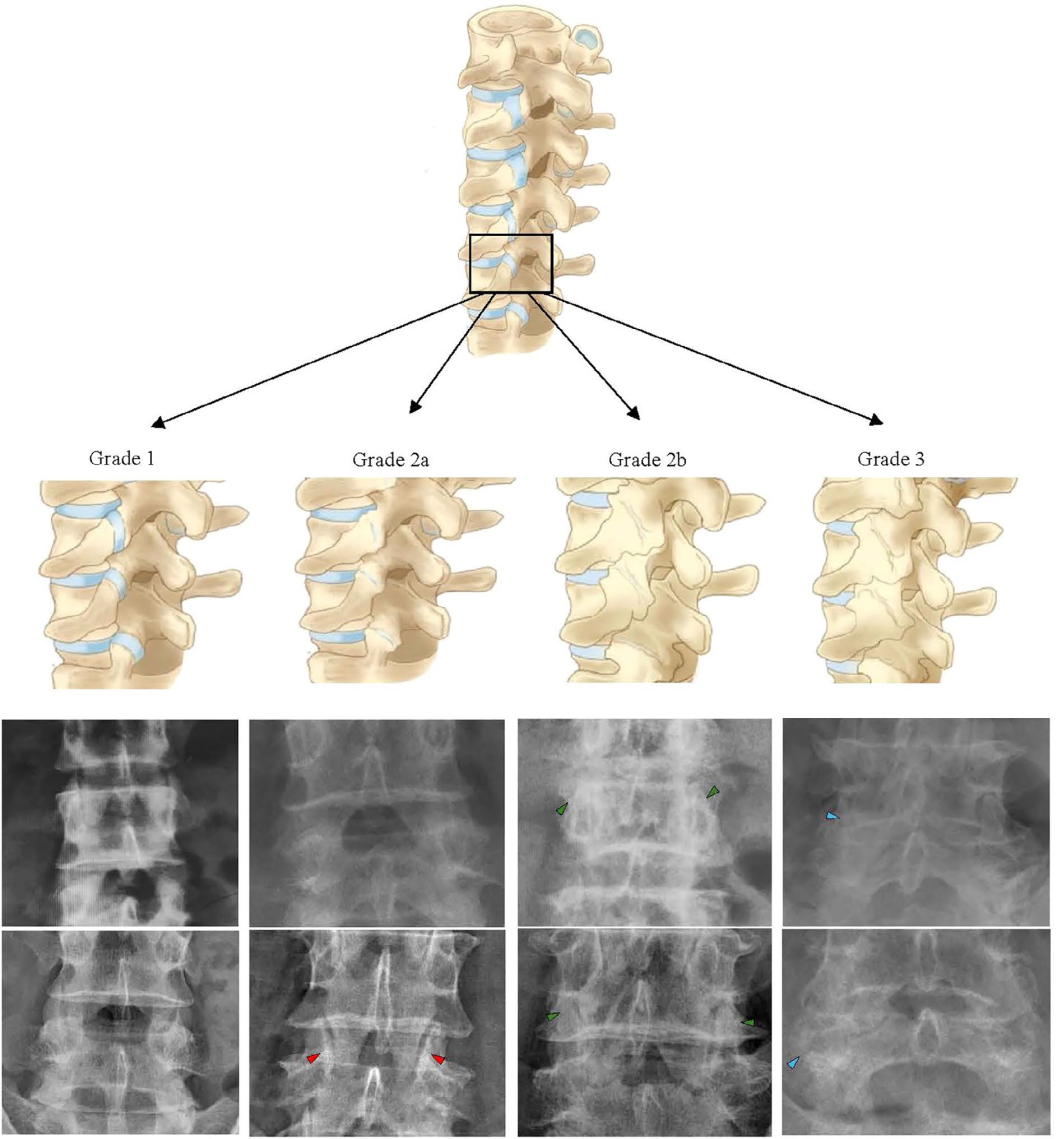
Example diagram based on the X‐ray grading system.

In this grading system, we have drawn from the osteoarthritis grading standards proposed by Kellgren‐Lawrence and made further modifications to better adapt them for the classification of lumbar facet joint degeneration. Therefore, we propose a new standard for grading of lumbar facet joint degeneration based on radiography. Grade I: The joint space is clearly visible (greater than 2 mm), and the surrounding bone structures are clear; Grade IIa: Joint space narrowing or obscurity (less than 2 mm) with no visible osteophyte formation around the facet joint, occasionally accompanied by subchondral bone sclerosis, while the surrounding structures remain relatively clear; Grade IIb: Joint space narrowing or obscurity (less than 2 mm) with significant osteophyte formation around the facet joint, making the surrounding structures difficult to distinguish; Grade III: The joint space disappears completely, and the normal structure around the facet joint is completely unrecognizable due to severe bone hyperplasia, which can be combined with changes or destruction at the margins of the facet joint space, or pseudocystic degeneration with the surrounding sclerotic zone (Figure 2) [20, 24].

For the included lumbar spine X‐ray images, the observer graded the degeneration of the L4/5 articular process joints. The evaluator needs to evaluate the joint processes on both sides simultaneously and ultimately take the most severe grade on one side as the level of lumbar facet joint degeneration in that segment. In addition, for calculation of the diagnostic performance, ≥ grade 2 facet joint degeneration was considered a positive finding.

### 2.6 Reference Standards

A senior radiologist and a senior orthopedic surgeon discussed and established a grading system for lumbar facet joints based on both X‐ray and MRI findings (Table 1). These two senior doctors then delivered lectures to two attending spinal surgeons (*Doctor A* and *Doctor B*) with 1–5 years of clinical experience, from different regions and hospitals, to explain the grading standards. Periodic assessments were conducted to ensure that the spine surgeons had a thorough understanding of the grading systems. Subsequently, these two spine surgeons were asked to independently evaluate and grade the X‐ray and MRI images of all included cases under a double‐blind protocol. Each imaging dataset was coded, de‐identified, and randomized to ensure blind assessment.

**TABLE 1:** The grading system for lumbar facet joints based on both MRI and X‐ray findings.

Analyzed finding and rating	Description
MRI classification (Weishaupt)	
0	Normal facet joint space (2–4 mm width)
1	Narrowing of the facet joint space (< 2 mm) and/or small osteophytes and/or mild hypertrophy of the articular process
2	Narrowing of the facet joint space and/or moderate osteophytes and/or moderate hypertrophy of the articular process and/or mild subarticular bone erosions
3	Narrowing of the facet joint space and/or large osteophytes and/or severe hypertrophy of the articular process and/or severe subarticular bone erosions and/or subchondral cysts
X‐ray grading	
1	The joint space is clearly visible (greater than 2 mm), and the surrounding bone structures are clear.
2a	Joint space narrowing or obscurity (less than 2 mm) with no visible osteophyte formation around the facet joint, occasionally accompanied by subchondral bone sclerosis, while the surrounding structures remain relatively clear.
2b	Joint space narrowing or obscurity (less than 2 mm) with significant osteophyte formation around the facet joint, making the surrounding structures difficult to distinguish.
3	The joint space disappears completely, and the normal structure around the facet joint is completely unrecognizable due to severe bone hyperplasia, which can be combined with changes or destruction at the margins of the facet joint space, or pseudocystic degeneration with surrounding sclerotic zone.

At the same time, the senior radiologist and senior orthopedic surgeon also grade the MRI and radiographs of the included cases. The difference between the grading results of the two senior clinicians finally determines a unique result through the discussion. The final result of the discussion between the two senior clinicians was called *Doctor C*.

One month after the initial evaluation, the same two attending spinal surgeons re‐evaluated and graded the radiographs of all included cases again at a separate workstation in the same manner.

### 2.7 Statistical Analysis

The minimum number of cases is confirmed by PASS 15 software. The intra‐ and inter‐rater reliability were evaluated by assessing the weighted kappa coefficient (κw). The intra‐ and inter‐rater reliability for grading of facet joint degeneration were evaluated as follows: 0–0.20, poor; 0.21–0.40, fair; 0.41–0.60, moderate; 0.61–0.80, good; and 0.81–1.00, excellent agreement. A *p* value of < 0.05 was considered statistically significant. Weighted kappa statistical analyses were performed using IBM SPSS Statistics version 26.0 (IBM Corp., Armonk, NY).

### 2.8 Ethical Review

The study protocol was approved by the Medical Institutional Review Board. All the methods were performed in accordance with the relevant guidelines and regulations.

## 3 Result

We included 103 patients in advance for pre‐classification. A total of 83 cases were evaluated by *Doctor A* and *Doctor B* to be completely consistent, 12 cases were consistent with 1 grade difference, and the other eight cases had an agreement with 2 or greater grade difference. The determined minimum number of cases required for statistical significance using PASS 15 software was 355. Subsequently, we continuously gathered data from November 2017 to September 2023, retrospectively collecting information from 443 patients. Ultimately, a total of 356 patients were included in the reliability analysis.

The senior radiologist and senior orthopedic surgeon graded the facet joints of the 356 included cases based on X‐ray and MRI. The final grading results were determined through discussion and consensus between these two clinicians and were called *Doctor C*. We believed that this result had certain reference significance and took it as the reference standard.

### 3.1 Inter‐Rater Reliability for MR Imaging

According to Weishaupt's MRI standards, inter‐rater reliability was evaluated for *Doctor A* and *B* with *Doctor C*. The level of inter‐rater reliability for complete agreement was good (κw = 0.792) for *Doctor A*, encompassing 88.76% (316 facet joints) of the cases. Agreement within 1 grade difference was observed in 96.07% of cases, representing an additional 7.31% (26 additional joints). Meanwhile, inter‐rater reliability was evaluated for *Doctor B* (κw = 0.869). *Doctor B* had 90.73% complete agreement (323 joints) and an additional 5.34% agreement with 1 grade difference (19 joints; agreement within 1 grade: 96.07%) (Table 2). For *Doctors A* and *B*, the weighted kappa coefficient for grading lumbar facet joint degeneration based on the MRI Weishaupt's standard was similar to the inter‐rater agreement (κw: 0.61) reported in previously published literature [15]. Therefore, we believe that these two surgeons have a solid theoretical and practical foundation in grading facet joint degeneration, which contributes to a more precise validation of the reliability of the grading method proposed later.

**TABLE 2:** Inter‐rater reliability for MRI of the facet joint degeneration for *Doctor A* and *B* with *Doctor C*.

	Doctor A&C	Doctor B&C
	Inter‐rater Reliability (Sessions 2)	Inter‐rater Reliability (Sessions 2)
Agreement	88.76%	90.73%
Agreement within 1 grade	96.07%	96.07%
Kappa (Kw)	0.792	0.869

### 3.2 Inter‐ and Intra‐Rater Reliability for X‐Rays

Based on the novel classification of lumbar facet joint degeneration on X‐rays, the inter‐rater reliability was also evaluated for both sessions for *Doctor A* and *B* with *Doctor C*. In the first session, *Doctor A* demonstrated good agreement (κw = 0.702, 78.65% agreement, 89.61% within 1 grade) as did *Doctor B* (κw = 0.714, 79.49% agreement, 91.01% within 1 grade). In the second session, *Doctor A* showed excellent agreement (κw = 0.847, 89.04% agreement, 96.07% within 1 grade) and excellent agreement for *Doctor B* (κw = 0.828, 88.20% agreement, 94.94% within 1 grade). Moreover, the level of intra‐rater reliability for complete agreement was excellent (κw = 0.843) for *Doctor A*, encompassing 88.76% (316 facet joints) of the cases. Agreement within 1 grade differences was observed in 95.51% of cases, representing an additional 6.75% (24 additional joints). Meanwhile, intra‐rater reliability was evaluated for *Doctor B* (κw = 0.836). *Doctor B* had 88.20% complete agreement (314 joints) and an additional 7.59% agreement with 1 grade difference (27 joints; agreement within 1 grade = 95.79%). In addition, we also calculate the inter‐rater reliability between *Doctor A* and *B* for sessions 2 (κw = 0.714), suggesting a good level of agreement between these two observers. We have summarized the above data in Table 3.

**TABLE 3:** Inter‐ and intra‐rater reliability for X‐ray of the facet joint degeneration.

	Doctor A			Doctor B			Doctor A&B
	Inter‐rater Reliability: *Doctor C* Session 1	Inter‐rater Reliability: *Doctor C* Session2	Intra‐rater Reliability	Inter‐rater Reliability: *Doctor C* Session 1	Inter‐rater Reliability: *Doctor C* Session2	Intra‐rater Reliability	Inter‐rater Reliability (Sessions 2)
Agreement	78.65%	89.04%	88.76%	79.49%	88.20%	88.20%	79.49%
Agreement within 1 grade	89.61%	96.07%	95.51%	91.01%	94.94%	95.79%	92.42%
Kappa (κw)	0.702	0.847	0.843	0.714	0.828	0.836	0.714

### 3.3 Reliability Between the Radiograph and MR Imaging

In order to verify the consistency between Weishaupt's MRI standard and our newly proposed X‐ray grading standard, we performed the following consistency tests. *Doctor A* demonstrated moderate agreement (κw = 0.459, 61.52% agreement, 86.52% within 1 grade) as did *Doctor B* (κw = 0.564, 68.82% agreement, 87.36% within 1 grade) (Table 4).

**TABLE 4:** Reliability between the radiograph and MR imaging for the facet joint degeneration.

	Reliability between MRI and X‐ray	
	Doctor A	Doctor B
Agreement	61.52%	68.82%
Agreement within 1 grade	86.52%	87.36%
Kappa (Kw)	0.459	0.564

These scores surpass the recommended threshold for an acceptable level of agreement (κw > 0.40) for grading lumbar facet degeneration [25].

## 4 Discussion

For decades, low back pain has been one of the most prevalent health issues globally, contributing significantly to years lived with disability [26, 27]. Numerous cases progress to CLBP, for which lumbar facet joint degeneration is a key contributor [28, 29]. In addition, we have observed a consensus on the classification of lumbar intervertebral facet joints using MRI and CT. However, both methods present drawbacks such as a high noise level, inconvenience, high cost, and operational complexity, limiting their widespread adoption [30, 31]. As X‐rays represent a cornerstone imaging tool in orthopedics, offering convenience, low radiation exposure, and affordability [32, 33]. In light of these findings, this prompts us to consider proposing a new grading system for lumbar facet joint degeneration based on X‐rays, which will serve as a supplement to Weishaupt and Pathria classification, enabling clinicians to have a more comprehensive understanding of lumbar facet joint degeneration and use a wider range of diagnostic tools to evaluate facet joint degeneration from multiple perspectives [34]. Therefore, based on the osteoarthritis grading system proposed by Professors Kellgren and Lawrence [35], and taking into account the unique anatomical structure and degenerative patterns of human spinal facet joints, we have developed a new grading system. The system aims to evaluate lumbar facet joint degeneration observed in radiological examinations.

In this study, we found that the grading system for lumbar spine X‐rays demonstrated acceptable levels of agreement both intra‐ and inter‐observers. Specifically, intra‐rater reliability reached excellent agreement (*Doctor A*: κw = 0.843; *Doctor B*: κw = 0.836), while inter‐rater reliability was good to excellent (κw ranging from 0.702 to 0.847). These results suggest that the grading system is robust and reproducible across observers with varying levels of experience. Additionally, the moderate agreement (κw = 0.459 for *Doctor A* and κw = 0.564 for *Doctor B*) between our X‐ray‐based system and the Weishaupt MRI standard underscores its diagnostic utility, particularly in resource‐limited settings where MRI is not readily available. All of the above scores exceeded the recommended threshold for acceptable consistency in grading lumbar facet joint degeneration (κw > 0.40) [25]. This also confirms the high repeatability and reliability of the proposed new grading method.

The reliability and diagnostic applicability of this grading system underscore its potential to serve as a practical and cost‐effective alternative to MRI in certain clinical scenarios. For example, patients with contraindications to MRI or those in resource‐constrained settings may benefit from this accessible and reliable X‐ray‐based approach [36]. Moreover, by incorporating facets of both anatomical and pathological changes, this system enables a nuanced evaluation of lumbar facet joint degeneration. In addition, the proposed X‐ray grading system's diagnostic utility lies in its ability to provide a quick and reliable assessment of lumbar facet joint degeneration [37]. This can enhance the ability of general orthopedic clinicians to detect and monitor degenerative changes, contributing to earlier diagnosis and timely management of CLBP. However, it is important to note that while the grading system adds value to the diagnostic process, it should not replace advanced imaging in complex cases.

This grading system emphasizes joint space narrowing, osteophyte formation, and subchondral sclerosis as primary features of degeneration [38]. By categorizing facet joint degeneration into distinct grades, clinicians can systematically describe the extent of degeneration, which may facilitate more standardized reporting and communication among healthcare professionals. Furthermore, this system may aid in identifying patients who require further advanced imaging or intervention. For example, patients with Grade 2b or Grade 3 degeneration may exhibit more severe anatomical changes, which could warrant closer evaluation using CT or MRI to confirm diagnosis and guide treatment planning.

In this study, we evaluated the potential clinical treatment applications of this grading system. It must be explicitly stated that the following discussion is not supported by direct data within this study but represents a reasonable extrapolation based on the authors' extensive clinical experience and the logical implications of the grading system. This exploration of clinical utility is intended to highlight the system's future application prospects rather than claim immediate clinical validation. (1) Surgical exposure and facet joint hypertrophy: Facet joint hypertrophy, particularly in Grade 2b and Grade 3 degeneration, poses significant challenges for surgical exposure during procedures such as transforaminal lumbar interbody fusion (TLIF) and posterior lumbar interbody fusion (PLIF). Preoperative evaluation of joint hypertrophy using this grading system may allow surgeons to anticipate the need for more extensive exposure, reducing intraoperative uncertainty [39]. Although full exposure of the facet joint is often inherent in these procedures, awareness of severe joint hypertrophy preoperatively may help optimize the surgical strategy, minimize unnecessary exposure, and potentially reduce blood loss [40]. (2) Pedicle screw placement: Narrowing of the joint space and significant osteophyte formation, as observed in Grades 2b and 3, can distort local anatomical landmarks critical for accurate pedicle screw placement [41]. By preoperatively identifying the severity of facet joint degeneration, surgeons may be better prepared to address these challenges intraoperatively. For example, severe degeneration may necessitate removal of hyperplastic bone or modification of the screw insertion technique to ensure proper alignment and stability. While this relationship requires further investigation through dedicated studies, we propose that the grading system has the potential to inform preoperative planning and improve procedural efficiency. Future studies should rigorously investigate these potential applications.

However, the study still has many limitations. The X‐ray‐based classification system for facet joints cannot be used independently to guide clinical surgical treatment. CT or MRI is still required as the gold standard for selecting the appropriate surgical approach. The primary purpose of this classification is to serve as a supplement to the Weishaupt and Pathria classifications, allowing clinicians to gain a more comprehensive understanding of lumbar facet joint degeneration and to use a broader range of diagnostic tools to assess lumbar joint degeneration from multiple perspectives. Furthermore, the correlation between the grading system proposed in this study and the success rate of pedicle screw placement, as well as postoperative complications, requires further research for validation. Finally, due to the fact that we did not use the Ferguson view during imaging and only relied on an AP view to visualize the patient's facet joints [42], there is potential for error when assessing the joint space at the L5/S1 facet joints. Future research needs to address these limitations to further refine and improve the classification system.

## 5 Conclusion

In the study, we propose a new grading system for lumbar facet joint degeneration based on X‐rays, which serves as a supplement to the Weishaupt and Pathria classifications. This system allows clinicians to gain a more comprehensive understanding of lumbar facet joint degeneration and utilize a broader array of diagnostic tools to assess degeneration from multiple perspectives. We believe this grading system can provide valuable insights into potential anatomical changes related to the facet joints, which can, in turn, inform modifications to surgical techniques and procedural steps.
